# The C-Type Lysozyme from the upper Gastrointestinal Tract of *Opisthocomus hoatzin,* the Stinkbird

**DOI:** 10.3390/ijms20225531

**Published:** 2019-11-06

**Authors:** Edward J. Taylor, Michael Skjøt, Lars K. Skov, Mikkel Klausen, Leonardo De Maria, Garry P. Gippert, Johan P. Turkenburg, Gideon J. Davies, Keith S. Wilson

**Affiliations:** 1 Structural Biology Laboratory, Department of Chemistry, The University of York, York YO10 5DD, UK; etaylor@lincoln.ac.uk (E.J.T.); Johan.Turkenburg@york.ac.uk (J.P.T.); Gideon.Davies@york.ac.uk (G.J.D.); 2 Novozymes A/S, Biologiens Vej 2, 2800 Kongens Lyngby, Denmark; mskj@novonordisk.com (M.S.); LaKS@novozymes.com (L.K.S.); MLKL@novozymes.com (M.K.); leonardo.demaria@astrazeneca.com (L.D.M.); GPGI@novozymes.com (G.P.G.)

**Keywords:** lysozyme, peptidoglycan cleavage, avian gut GH22, crystal structure

## Abstract

Muramidases/lysozymes are important bio-molecules, which cleave the glycan backbone in the peptidoglycan polymer found in bacterial cell walls. The glycoside hydrolase (GH) family 22 C-type lysozyme, from the folivorous bird *Opisthocomus hoazin* (stinkbird), was expressed in *Aspergillus oryzae*, and a set of variants was produced. All variants were enzymatically active, including those designed to probe key differences between the Hoatzin enzyme and Hen Egg White lysozyme. Four variants showed improved thermostability at pH 4.7, compared to the wild type. The X-ray structure of the enzyme was determined in the apo form and in complex with chitin oligomers. Bioinformatic analysis of avian GH22 amino acid sequences showed that they separate out into three distinct subgroups (chicken-like birds, sea birds and other birds). The Hoatzin is found in the “other birds” group and we propose that this represents a new cluster of avian upper-gut enzymes.

## 1. Introduction

**Figure 1:**
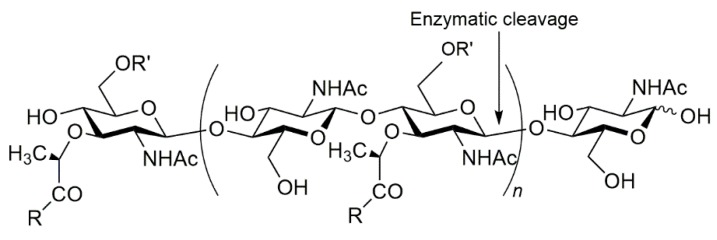
The cleavage site of the cell wall glycan by lysozyme/muramidase.

Peptidoglycans are unique to prokaryotic organisms and consist of a glycan backbone of muramic acid and glucosamine (both N-acetylated), cross-linked with peptide chains. In Gram-positive bacteria (e.g., *Staphylococcus aureus*) the glycan backbone is highly cross-linked, while it is only partially cross-linked in Gram-negative bacteria, such as *Escherichia coli*. The cross-linking amino acid chain contains L-alanine, D-glutamic acid, meso-diaminopimelic acid, and D-alanine in *E. coli,* or L-alanine, D-glutamine, L-lysine, and D-alanine, with a five-glycine interbridge between tetrapeptides, in the case of *S. aureus* [1]. The unique composition of both the carbohydrate polymer and the peptide cross-linker means that only specialised enzymes can hydrolyse peptidoglycans. Lysins (amidases, lysozymes/muramidases and peptidases) are such specialised bio-molecules. The term lysozyme, or muramidase, is broadly used to describe the enzymes that cleave the β-1,4-glycosidic bond between *N*-acetylglucosamine (NAG) and *N*-acetylmuramic acid (NAM) (or vice versa) in the carbohydrate backbone of peptidoglycan, Figure 1.

In nature, the β-1,4 bonds of peptidoglycan are cleaved by a structurally diverse set of enzymes. Lysozymes/muramidases (EC 3.2.1.17) are found in several glycoside hydrolase families in the Carbohydrate-Active enZYmes Database (CAZy, www.cazy.org [2]), including GH18, 19, 22, 23, 24, 25, 73 and 108, some of which, such as GH25, remain largely uncharacterized biochemically. While the first three families have very low sequence identities, they do have some common structural features, consisting of a constant core of two helices and a three-stranded β-sheet that accommodates the substrates in the inter-domain cleft [3]. Higher organisms typically have enzymes from several GH families, e.g., *Gallus gallus* has at least three from GH18, GH22 and GH23. An excellent review of “Lysozymes in the animal kingdom” [4] summarises a wealth of information on the enzymes, and their classification into subfamilies—lysozymes C (chicken-type, the archetypal lysozymes), G (goose-type) and I (invertebrate). Subfamilies C and I are both grouped in CAZy family GH22, while G is in GH23.

CAZy has over 700 entries for GH22, almost all from Eukaryota, but the 3D structure has only been determined for about 25 species. The most well-known is the C-type lysozyme from *G. gallus* (chicken), commonly called Hen Egg White Lysozyme (HEWL), which is almost synonymous with lysozyme. The deposited structures are dominated by the enzymes from chickens and *Homo sapiens*. While the overall amino acid sequences identity is quite low, the structural similarity of the C-type lysozymes is very high. A partial explanation for this is assumed to be due to the four conserved disulphide bridges, that ensure a compact and rather rigid 3D arrangement.

The structure of HEWL revealed the GH22 fold [5] to be a α + β motif, made up of five α-helical regions and five containing β-strands, with two catalytic groups, Glu35 and Asp52. The active site consists of six subsites (originally termed A, B, C, D, E and F, but now more generally named −4, −3, −2, −1, +1 and +2) [6], which bind up to six consecutive sugar residues. The glycosidic bond between the N-acetyl muramic acid (NAM) at subsite −1, and the N-acetyl -glucosamine (NAG) at subsite +1, is weakened by steric distortion of the sugar ring in subsite −1, and is the target of the hydrolytic cleavage. In 2001, experimental evidence for the correct working mechanism of HEWL was finally established [7], with the hydrolysis of the β-(1,4)-glycosidic bond occurring through a double displacement reaction. This mechanism is believed to apply to all members of this very broad class of enzymes.

HEWL has over 25 years of recorded use in wine and cheese making [8], and, classically C-type GH22 lysozymes have been known to act as antimicrobials, or at least as microbial growth inhibitors [9,10]. In addition, some mammalian (typically ruminant) GH22s have been proposed to have a digestive role in the stomach, where they could degrade bacteria after the front-gut fermentation process [11]. More recently, lysozymes have been proposed to modulate the bacterial flora and to digest bacterial cell wall debris, thereby affecting the immune system [12].

If selected lysozymes could be expressed in a heterologous host, suitable for industrial production, these could be used in applications where peptidoglycans are present and their elimination would be useful (such as biofilms, washing and nutritional supplements). For a long time, the literature indicated that lysozymes were difficult to express in such hosts. In particular, the group of David Archer at the University of Nottingham published more than 10 papers on the heterologous expression of HEWL and human lysozyme in *Aspergilli* [13] and, indeed, in *Pichia,* albeit with limited yields being obtained. Furthermore, the lack of gastric stability has been a hindrance for commercializing HEWL as an animal feed additive.

With the aim of expressing a digestive lysozyme in a suitable fungal host, the literature was scanned for a GH22 C-type lysozyme with high stability at low pH (gastric conditions). Earlier reports had indicated the extraordinary stability of the digestive lysozyme from *Opisthocomus hoazin* at low pH [14], and the corresponding cDNA sequence (Genbank entry AAA73935.1) had been published [15]. *O. hoazin*, known as the Hoatzin, or stinkbird, lives in parts of the rainforest in South America. The Hoatzin is unique in being the only known bird with crop fermentation in the foregut [15,16]. A recent review of avian crop function covers the importance of digestion in bird species [17]. In some respects, digestion in the Hoatzin gut seems more like that of ruminants than other folivorous birds, and is derived from the morphological and microbiological environment in the digestion tract [14]. Both ruminants and Hoatzins express high levels of gastric lysozyme and use fermentation in their foregut. This enables the Hoatzin to take advantage of energy from both the cellular content and the cell wall polysaccharides of hydrolysed bacteria [18]. Lysozyme is an important element of the Hoatzin’s digestion system, wherein the digestive tract of the Hoatzin can also be found. Based on the predicted amino acid sequence, HEWL and *O. hoazin* lysozyme (henceforth *Oh*Lys) have very different isoelectric points. As a result, quite different properties for their selectivity might be expected.

We here report the successful cloning and expression in *Aspergillus oryzae* (NCBI:txid90341) of a synthetic gene, corresponding to *Oh*Lys. Mutational studies were applied to modify the activity and/or stability of *Oh*Lys. In addition, we have determined the crystal structure of the apo enzyme and of complexes with reaction products (chito-oligosaccharides). This is the first structure of an avian GH22 lysozyme/muramidase outside the chicken-like sub-group.

## 2. Results

### 2.1. Expression of OhLys and Variants in Aspergillus

HEWL, the best characterized GH22, shows a surprising level of promiscuous chitinolytic activity [19]. As the *Aspergillus* fungal cell wall consists mainly of chitin, expression of an enzyme with chitinase activity may be expected to counter-select high-level expressing transformants. Indeed, Archer et al. previously concluded that proteolysis occurs in HEWL between Gly49 and Ser50 when it is expressed in *A. niger* [20]. In contrast, our results show that *Oh*Lys can be expressed at a reasonable level (about 1 g/L) in *A. oryzae*, with about 30 variants being produced and shown to be active in the turbidity assay.

### 2.2. Crystal Structure of the Apo Enzyme and Its Chitotriose Complex

**Figure 2:**
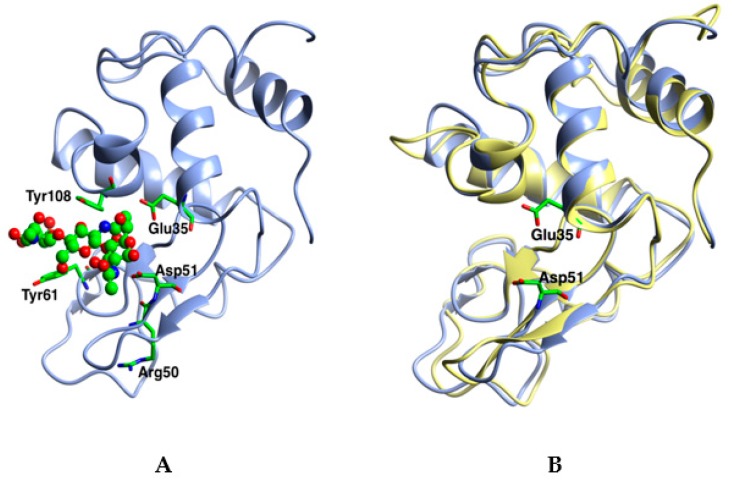
The 3D structure of *O. hoazin* lysozyme (*Oh*Lys). (**A**) *Oh*Lys shown as ribbons with the chitotriose from the complex as ball and stick binding in sites -1 to -3. The position of the catalytic Glu35 and Asp51 (cylinders) are taken from the apo structure. Three key residues which were subsequently mutated to resemble those in Hen Egg White Lysozyme (HEWL) are also shown as cylinders: Arg50, Tyr61 and Tyr108. (**B**) Superposition of the *Oh*Lys (blue) on HEWL (yellow).

Crystals of the apo enzyme belong to the orthorhombic space group P2_1_2_1_2_1_ with one molecule in the asymmetric unit, Table 1. As expected, *Oh*Lys has a typical GH22 lysozyme fold (Figure 2). The chain was traced from Glu1 through to Cys126, with a glycerol from the cryoprotectant, four Cl^−^ ions and 200 water molecules. There are four disulphide bridges (Cys6–Cys126, Cys30–Cys114, Cys63–Cys79 and Cys75–Cys93) in the structure.

The GH22 fold is highly conserved over a range of organisms, as can be seen in Table 2, where the r.m.s. difference in Cα position over between 112 and 125 residues is between 0.79 and 1.4Å, from rainbow trout to mouse. The 3D structure is highly conserved; the chains are of very similar lengths in all species, with almost no deletions or insertions. The structures show a remarkably high level of similarity, greater than might be expected for the sequence identity, with the r.m.s. difference in Cα positions showing little correlation with the evolutionary tree for the GH22 lysozymes discussed below. In part, this likely reflects the conserved set of disulphide bridges in these enzymes. The only variation is in the so-called calcium loop, at the bottom of the structure in Figure 1, with residues in the range 45–51, which is displaced in a couple of the structures. This loop is occupied by a sodium ion in several deposited PDB files. The last two GH22 structures in the Table, from bivalves, show somewhat more extensive differences in several loops, hence the reduced number of equivalent Cα atoms.

**Figure 3:**
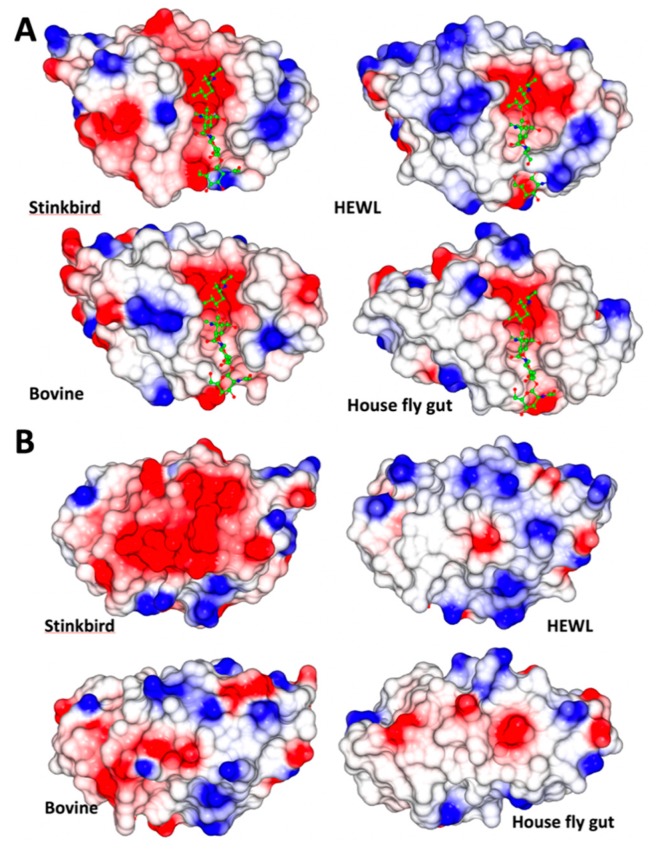
Electrostatic surface charge (red: negative and blue: positive) for *Oh*Lys, HEWL, Bovine gut lysozyme and house fly gut lysozyme, calculated at pH 7.0, within CCP4mg. (**A**) viewed from the active site side of the enzymes, (**B**) from the opposite side. The single clear observation is that the rear (**B**) of *Oh*Lys carries a substantially higher negative charge. The structures were superposed using the Gesamt option in CCP4mg. The ligand is taken from the *Oh*Lys complex.

The overall charge of *Oh*Lys differs somewhat from that of other C-type lysozymes (Table 3). This increase in negative charge of *Oh*Lys is evident in the surface electrostatics of the enzyme (Figure 3). While the significance of this is not clear, it should be noted that the peptidoglycan substrate is also a charged molecule, and that peptidoglycan from different bacterial sources have different pI. So, charge interactions between the enzyme and the substrate at working pH are probably functionally important.

Co-crystallisation of the inactive variants of the enzyme with chito-oligosaccharides was partially successful. Screening for ligand complexes was carried out with the supposed inactive mutants, E35A and D51A. E35A itself crystallised readily in INDEX screen no. 7 (Hampton Research) in Falcon 24 well plates, producing large, well-diffracted crystals, while it was not possible to obtain crystals from the D51A mutant. Co-crystallisations of E35A were set up with chitobiose, chitotriose, chitotetraose, chitopentaose and chitohexaose, and were successful with chitobiose, chitotetraose, and chitohexaose. The resulting electron density clearly confirmed the Glu to Ala mutation.

**Figure 4:**
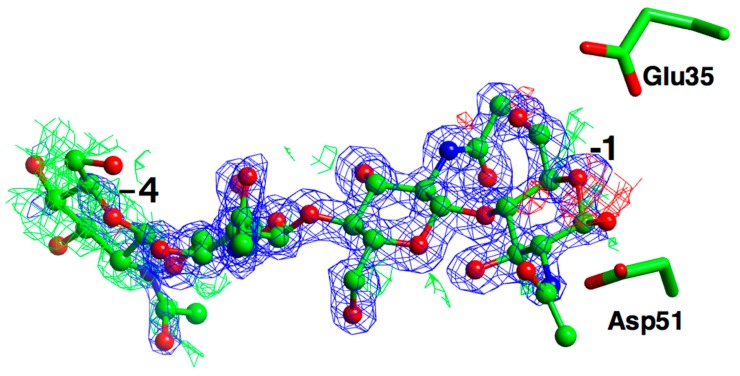
Electron density maps for the ligand from the crystal co-crystallised with chitohexaose. The REFMAC maximum likelihood weighted map, contoured at 1 σ, is shown in blue, the difference map, contoured at 2.5 σ in green (positive), and red (negative), with phases calculated prior to the incorporation of any ligand atoms in refinement. The catalytic residues are superposed from the apo structure.

The electron density for the crystal soaked in chitohexaose is shown in Figure 4, which shows excellent density for the −2 and −3 subsite sugars, and good density for −1, with poorly ordered density for −4, with a significant difference in density around this subsite. Unfortunately, there is no density in the +1 subsite—a key aim of the experiment had been to observe sugar bound across the point of catalysis between −1 and +1. It is evident that the mutant retains a sufficient level of residual activity to hydrolyse; at the high protein concentrations of a prolonged crystallization, the chitohexaose substrate to a mixture of the two, three and four membered sugars. The density for the chitotetraose ligand is essentially identical to this, while the chitotriose only shows binding in sites −1 to −3. Sites +1 and +2 do appear to be accessible in the structure, and binding of non-hydolysable chitohexaose analogues, for example, with sulphur replacing the glycosidic oxygens, might prove successful if such ligands should become available.

### 2.3. Position of OhLys in the GH22 Family Tree: the Avian Gut Enzymes

**Figure 5:**
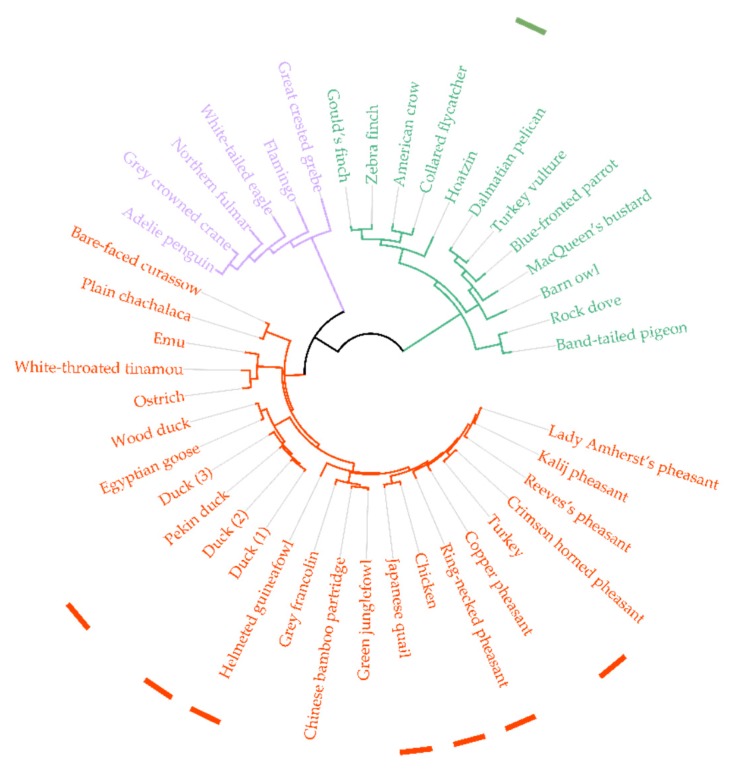
Phylogenetic tree of avian GH22 lysozymes, marked with different colours for different types of birds (red: chicken-like, violet: seabirds, and green: “other birds”/“upper gut”). Truncated sequences were omitted from the analysis and sequences that are more than 98% identical are shown as one entry. Known 3D structures in the PDB are indicated by coloured squares (green: *Oh*Lys and red: pdb). A list of sequence identifiers (common name, scientific name, database ID and cluster sizes can be found in Supplementary Table S1.

**Figure 6:**
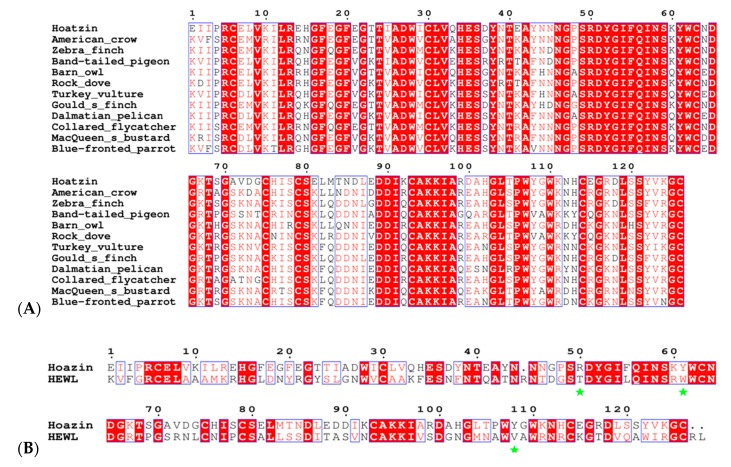
(**A**) Sequences of the proposed avian gut set of GH22 lysozymes: the SWISSPROT/PDB codes and full species names are listed in Supplementary Table S1. (**B**) For comparison, the alignment of *Oh*Lys with HEWL. The sequence numbers correspond to *Oh*Lys. Conserved residues are highlighted in red; conservative changes are shown in red text. There is a single deletion at residue 45 in the *Oh*Lys sequence, and a loss of two amino acids at the C-terminus. The Hoatzin sequence is that reported from the gene sequencing [15], and does not include the two amino acid insertion after residue 1 in the *Oh*Lys variant. Figure produced using ESPrpt [22]. Three key differences are indicated by * (R50/T51, Y61/W62 and Y108/V109): each of these residues was mutated from its *Oh*Lys amino acid to that in HEWL, as part of the mutational studies. The first two of these are totally conserved in the avian gut family in (**A**), the latter is either Y or V.

A bioinformatic analysis of GH22 amino acid sequences shows that vertebrates and invertebrates separate into different subgroups, and in the vertebrates, avian species separate out into three distinct subgroups (chicken-like birds, sea birds and other birds), represented in a tree (Figure 5). *Oh*Lys is found in the “other birds” group. We propose that this cluster represents avian upper-gut enzymes and that there is a physiological reason for this differentiation; one observation is that seabirds and “other birds” feed their chicks, while the chicken-like group does not. In the chicken-like group, high amounts of lysozyme are found in the egg. There is, at present, no conclusion as to which avian lysozymes are digestive gut/crop enzymes. While, for a few (Hoatzin, for example) it has it been firmly established that the lysozyme functions in the crop, it is of note that the avian “upper gut” group (including the Hoatzin, Zebra finch, dove and others, Figure 5) lies on a separate branch of the tree from the avian-egg enzymes, and has conserved changes relative to HEWL. A number of the produced variants addresses the conserved differences between HEWL and the “upper gut” group (for example, Arg50, discussed below). We propose that the “upper gut” lysozymes are utilized differently in these species and that this division will allow for further work regarding the digestive system in birds (and other organisms) by comparing their GH22s. The sequences of the proposed avian gut lysozymes are aligned in Figure 6A and the Hoazin sequence compared to HEWL in Figure 6B. 

### 2.4. Properties of OhLys and Variants

#### 2.4.1. The Extended Set of OhLys Variants

In these variants, a kexin-like protease B (kexB) site was introduced between the signal and the mature region, inserting a lysine and arginine (*1aK *1bR) between residues Glu1 and Ile2 of the wild type sequence. The variants listed in Table S2 were originally designed to probe a number of properties, as shown, and, while these were not all characterised in detail, the results of some mutations are briefly described below.

A selected set of variants was produced in larger amounts, purified and tested for pH optimum, and thermostability, T_m_, was measured using Nano differential scanning fluorimetry (Table 4) (all variants derived from *Oh*Lys-KexB). The data show that R50T is destabilized at both pH 4.7 and 8.0, while D90A and Y108V are stabilized, especially at pH 8.0. In summary, Arg at position 50 makes *Oh*Lys more stable, while the D90A and Y108V changes to HEWL residues make the enzyme more alkaline-stable. The latter pair would make these two constructs good candidates for applications requiring high pH stability.

#### 2.4.2. Retention of Activity of OhLys at Low pH

The pH activity profile on peptidoglycan from *Micrococcus lysodeikticus* was measured for the wild-type and a set of mutants. It cannot be guaranteed that this corresponds directly to the activity on the peptidoglycan found in the real in vivo situation. The pH optimum of the WT *Oh*Lys is 4.4, while the KexB insertion increases this to ~5.0. The optimum remains at 5.0 for the other mutants, only reverting to ~4.4 for the R50T and Y61W variants. These values should be compared with those reported earlier for a set of digestive and more typical lysozymes [11], which showed that the optima for three ruminants were in the range 4.5–5.0, while those for the typical chicken and pig lysozymes had a broad peak between 6 and 8, with the leaf-eating monkey lysozyme around 5.2. This supports the acquisition of a low pH optimum for *Oh*Lys, as a crop digestive enzyme, which has evolved independently of the ruminant set.

**Figure 7:**
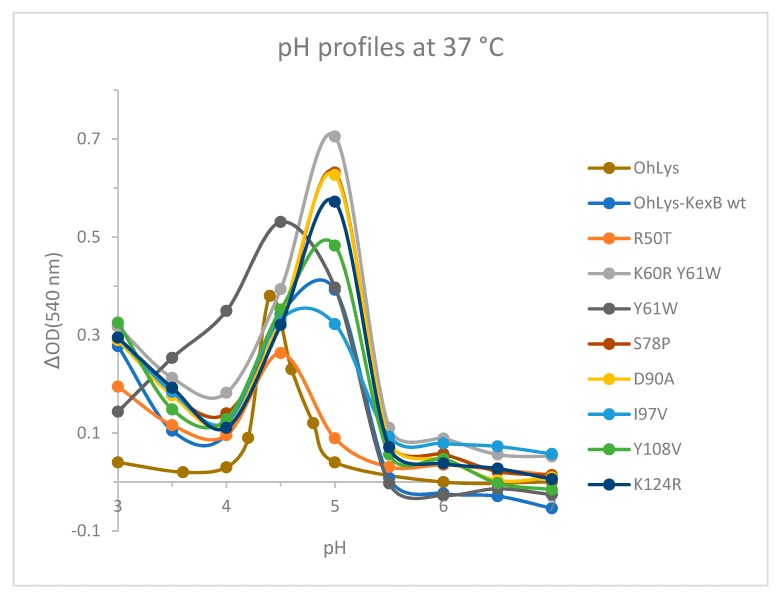
The pH profiles of wild-type *Oh*Lys, the *Oh*Lys-KexB wt, and a selected set of variants, all derived from *Oh*Lys–KexB.

**Figure 8:**
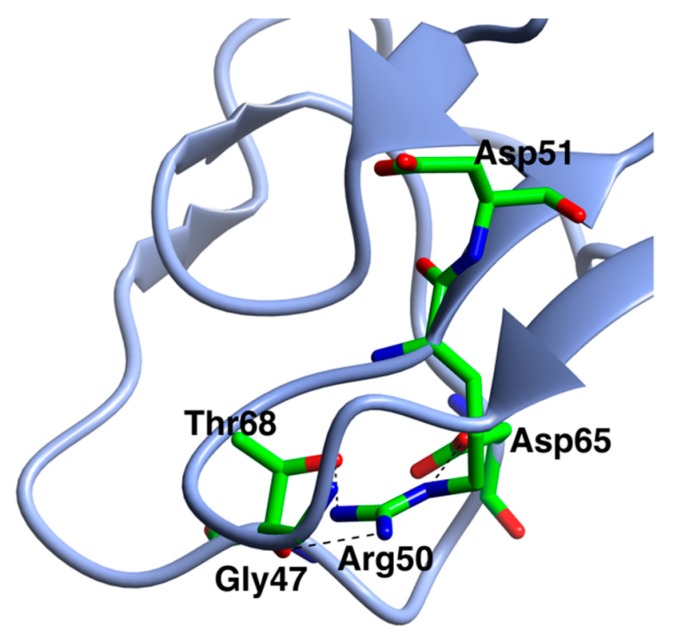
The position of Arg50 adjacent to the catalytic Asp51 in the sequence, showing the residues with which its side chain forms H-bonds, shown as dashed lines.

From the pH curves of the variants (Figure 7) it was clear that almost all constructs with the kexB insertion had a shift in the pH optimum, from pH 4.4 to about pH 5. It seems likely that this is caused by the additional positive residues, which increase the pI. Five of the seven variants had higher activity than the WT, while two had lower. The two variants with the Y61W mutation had the highest activity compared to HEWL. Variant R50T had significantly lower activity and stability than the WT, and it seems that activity at pH 5 has almost been eliminated. Arg50 is adjacent to the catalytic Asp51 in the chain (Figure 8) and its sidechain is hydrogen-bonded to the sidechains of Asp65 and Thr68, and to the backbone O of Gly47. Both Asp65 and Thr68 are in the loop region from Cys63 to Cys79. Removal of the Arg50 sidechain will remove these hydrogen bond possibilities and could, therefore, lead to a slight rearrangement of this part of the structure, leading to a new position of the catalytic Asp51 and decreased stability of this part of the enzyme.

The two variants with the Y61W mutation had the highest activity. Kumagai and co-workers mutated Trp62 to His in HEWL, and observed an altered substrate-binding mode [23]. In *Oh*Lys, the corresponding residue is Tyr61, which is found frequently among GH22s, with the Trp almost exclusively occurring in HEWL. Variant R50T had significantly lower activity and stability.

**Figure 9:**
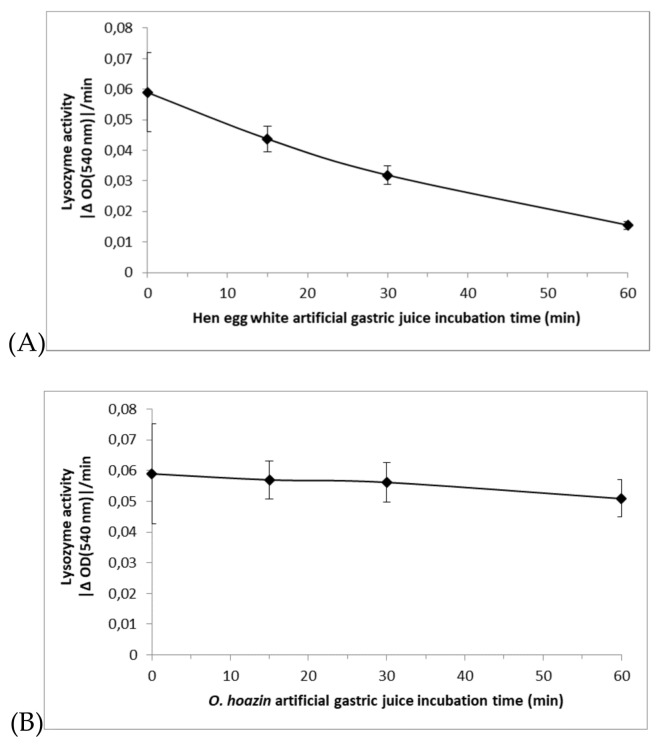
Activity of (**A**) HEWL and (**B**) *Oh*Lys measured by the turbidity assay after incubation, as described above under “In vitro stability of OhLys vs. HEWL under gastric conditions”. *Oh*Lys retains a high level of activity after one hour (close to 100%), while that of HEWL has dropped substantially. These results refer to WT *Oh*Lys.

The low pH optimum and pepsin resistance reported earlier for *Oh*Lys (Kornegay et al., 1994 and Ruiz et al., 1994) were confirmed for the enzyme expressed in *A. oryzae*. A comparison of activity after incubation under gastric conditions, between HEWL and *Oh*Lys, is shown in Figure 9.

## 3. Discussion

The major aim of this study, which was successfully achieved, was to establish a possible alternative lysozyme to HEWL for application in animal feed, where the low gastric stability of HEWL posed a major problem for survival in the stomach, so the enzyme could act in the intestine (where most peptidoglycan is present, either dead or alive). The Hoatzin had been previously described as having a gastric stable GH22 lysozyme, and, in addition, it is a creature in which the genome had been sequenced (although more common now, this information is still only available for a limited number of birds). Expression in a micro-organism is essential for an economically viable commercial product, so successful overexpression in an *Aspergillus oryzae* host presents an important advance in this, and enables protein engineering for modifying properties, such as pH-optimum or T-stability. The results confirmed that the enzyme showed considerably better gastric stability, key for its potential application, compared to that of HEWL, reported previously in [11]. The pH optimum of wild type *Oh*Lys was 4.5. While the chicken feed application has been superseded by the development of a GH25 muramidase into a successful commercial product (Balancius^TM^ from DSM nutritional products, Kaiseraugst, Switzerland) [24,25], the observations made for *Oh*Lys could be useful in other applications.

With regard to the variants, possibly the most important observation is that a range of mutations could be made and expressed quite easily, while retaining stability and catalytic activity. The quality of the experimental analysis of the properties of the mutants was limited by the OD assay, which, while being a well-established lysozyme analytical tool, is not highly reproducible. The introduction of the KexB mutation, involving the insertion of two residues *1aK *1bR, increased the pH optimum by one pH unit, and this remained essentially unchanged in the set of mutants based on this variant. The kexB mutation was not successful in allowing removal of the N-terminal Glu. Finally, some mutations appeared to have higher specific activity, and four variants had higher thermal stability.

Bioinformatic analysis of avian GH22 amino acid sequences shows that they separate into three distinct subgroups (chicken-like birds, sea birds and other birds). *Oh*Lys is found in the “other birds” group, and we propose that this cluster represents avian upper-gut enzymes and that there is a physiological reason for this differentiation.

The structure presented here is the first to be determined for the avian upper-gut subset of GH22 lysozymes, and is, as expected, very similar overall to other C-type lysozymes. The close similarity in the 3D structure for this family—more than might be expected for the sequence similarity—is assumed to be at least in part due to the disulfide bridges. Indeed, it is notable that the enzymes can be different, with, for example, low sequence ID and different pH-optima, despite having such similar structural frameworks. The binding of the ligand in subsites −4, −3, −2 and −1 is typical for this family. Unfortunately, the “inactive” mutant retained sufficient activity to degrade longer oligosaccharide substrates, and so the complexes did not span the −1 and +1 sites as planned. However, the long active site cleft is accessible to ligand in the crystals, which may prove beneficial in future.

In summary, we present the first structure and a ligand complex of a digestive lysozyme from a new sub-group of GH22s from the avian upper gut. *Oh*Lys can be expressed in a production-relevant host and protein engineering can be performed to optimize the performance. 

## 4. Materials and Methods

### 4.1. Cloning, Expression and Purification of OhLys

#### 4.1.1. Heterologous Expression of OhLys in *Aspergillus oryzae*

According to the published Genbank entry (AAA73935.1, [15]) genomic DNA was extracted from a frozen hoatzin tissue sample provided by the Louisiana State University Museum of Vertebrate Zoology collection *(Opisthocomus hoazin (*GenBank^®^ accession number L36032) and the sequence determined. This was used as a starting point to design an artificial coding region (CDR), which was adapted to *Aspergillus oryzae* codon usage. The CDR was amplified by PCR, and cloned into an expression vector using InFusion cloning. Resulting clones were sequenced on both strands and transformed into an *A. oryzae* (NCBI:txi 90341) expression host. Transformants were grown for four days in yeast extract/Peptone/Mannitol 5 g/3 g/25 g per liter supplemented media in a microtiter plate, and the expression in the resulting supernatants was evaluated by SDS-PAGE. The best transformant was spore purified twice.

#### 4.1.2. Fermentation and Purification

The transformant was fermented in shaker flasks at 30 °C for 72 h. Culture broths were filtered through filtration cloth, and, subsequently, through a 0.2 µm filtration unit (Nalgene, Thermo Fisher Scientific, Waltham, MA, USA) to remove the *Aspergillus* host. Solid NaCl was added to a final concentration of 200 mM and the pH was adjusted to pH 5.5 with 20% CH_3_COOH. The adjusted enzyme solution was applied to a SP-sepharose FF column (GE Healthcare (Brondby, Denmark)), which was equilibrated in 50 mM CH_3_COOH/NaOH, 200 mM NaCl, pH 5.5. The column was thoroughly washed with an equilibration buffer to remove loosely bound protein. The enzyme was eluted using a linear NaCl gradient (200 mM to 1000 mM) in 50 mM CH_3_COOH/NaOH, pH 5.5 over five column volumes. *Oh*Lys eluted as a single peak, and the purity was analysed by SDS-PAGE. The resultant protein solution was concentrated to 26 mg mL^−1^ and buffer exchanged into 25 mM HEPES pH 7.5, using a Vivaspin ((Sartorius, Goettingen, Germany) 10-kDa cut-off concentrator. Edman degradation on the purified protein indicated an N-terminus, corresponding to the expected (EIIPRCELVK-), and intact molecule mass spectrometry gave a molecular weight of 14260.4 g/mol (theoretical value 14,260.9 g/mol).

### 4.2. Expression of OhLys Variants

In the first variant, a kexin-like protease B (kexB) site was introduced between the signal and the mature region, inserting a lysine and arginine (*1aK *1bR) between residues Glu1 and Ile2 of the wild-type sequence. The aim had been to remove the N-terminal glutamate, to avoid pyro-glutamate formation, but unfortunately no cleavage was observed with kexB, and the result was a construct with an N-terminal sequence of E-1 K0 R1 I2 I3 P4 R5 C6 E7 L8 V9- (the residues are numbered from -1, so as to retain the normal mature sequence for the bulk of the chain), instead of the hoped for IIPRCELV-. This is henceforth referred to as *Oh*Lys-kexB.

Subsequent variants were made by established methods (see WO 2012/035103 [26]) starting from the *Oh*Lys-kexB variant, rather than the true wild-type. Variants were transformed into an *A. oryzae* host and the expression estimated by SDS-page. Where appropriate, the activity was evaluated using a turbidity assay, with *Micrococcus luteus* as substrate (see below). The variants fell into two groups, described in the following sections. The first aimed to prepare enzymatically inactive constructs for co-crystallisation with ligands, and the second group targeted changing a number of properties, as listed in Table S1, including three aimed at key differences between *Oh*Lys and HEWL: R50T, Y61W and Y108V.

#### 4.2.1. Inactive Catalytic Site Variants

Based on the *Oh*Lys-kexB variant, four variants (E35A, E35Q, D51A and D51N) of the catalytic residues E35 and D51 were produced and purified with the methods described above. The aim was to express an inactive enzyme for co-crystallisation with ligand. 

#### 4.2.2. The Extended Set of Variants

An extensive set of variants was created, all starting from the *Oh*Lys-kexB variant, and are briefly summarised in Supplementary Table S1. A key observation was that that all these variants were shown to retain enzyme activity using the turbidity assay below, supporting the robustness of the *Oh*Lys fold.

#### 4.2.3. Preparation of *Micrococcus Lysodeikticus* Substrate

Before use, *M. lysodeikticus* cells were resuspended in citric acid/phosphate buffer pH 4.4 in a concentration of 0.5 mg cells/mL and the optical density (OD) was measured at 540 nm. The cell suspension was adjusted so the cell concentration equaled an OD_540_ of 1.0, and the adjusted cell suspension was stored cold before use. Resuspended cells were used within 4 h.

#### 4.2.4. Turbidity Assay of Activity

The OD-drop assay measures lysozyme activity through a reduction in OD (light scattering) caused by cell lysis (cell wall hydrolysis), as described in many papers on HEWL [11,27]. Here, the activities of *Oh*lLys and variants were determined by measuring the decrease (drop) in absorbance/optical density of a solution of resuspended *Micrococcus lysodeikticus* ATTC No. 4698 (Sigma-Aldrich M3770), measured in a spectrophotometer at 540 nm (https://www.sigmaaldrich.com/technical-documents/protocols/biology/enzymatic-assay-of-lysozyme.html and [28]).

#### 4.2.5. Measurement of Lysozyme Activity in the Turbidity Assay

The lysozyme sample was diluted to a concentration in the range 100–200 mg enzyme protein/L in citric acid/phosphate buffer pH 4.4 and kept on ice until use. In a 96 well microtiterplate (Nunc), 200 μL of the substrate was added to each well, and the plate incubated at 37 °C for 5 min in a VERSAmax microplate reader (Molecular Devices, Wokingham, United Kingdom). Following incubation, the absorbance of each well was measured at 540 nm (start value). To start, the activity measurement 20 μL of the diluted lysozyme samples was added to the 200 μL substrate in each well and kinetic measurement of absorbance at 540 nm was initiated for a minimum of 30 min, up to 24 h, at 37 °C. The measured absorbance at 540 nm was monitored for each well, and over time, and a drop-in absorbance was taken as the drop-in lysozyme activity. To compare results from the turbidity assay, the samples to be compared were tested in the same experimental run, using the same buffer and substrate batch. 

### 4.3. Biophysical Properties of OhLys and Variants

#### 4.3.1. pH Optimum of *Oh*Lys

pH activity curves between pH 3 and 7 were recorded using the turbidity assay described above, but with the pH of the citrate/phosphate buffer adjusted to the relevant value. All samples were diluted to an enzyme concentration of 50 µg/mL. The wild type enzyme, without the KR insertion, had a pH optimum of pH 4.4.

#### 4.3.2. In Vitro Stability of *Oh*Lys vs. HEWL under Gastric Conditions

*Oh*Lys and HEWL were incubated for 0, 15, 30 and 60 min in artificial gastric juice (HCl pH 2, 1 mg/mL pepsin, 0.1 M NaCl) at 37 °C, and their activity determined using the turbidity assay described above.

#### 4.3.3. Thermostability of Variants Using Nano Differential Scanning Fluorescence

Nano Differential Scanning Fluorescence (NanoDSF) was performed with a Prometheus NT.48 instrument (NanoTemper Technologies GmbH, München, Germany). Purified *Oh*Lys variants (in either 250 mM Na-acetate, pH 4.7, or 20 mM tris(hydroxymethyl)aminomethane, pH 8.0) were loaded into nanoDSF standard grade capillaries (NanoTemper Technologies GmbH; catalogue number PR-C002) through capillary action. Three capillaries were filled for each sample. The capillaries were then placed into the instrument (up to 48 single capillaries can be loaded in a single run) and the laser intensity required for optimum signal generation was determined. The samples were run with the following experimental setting: temperature slope 2 °C/minute, start temperature 20 °C and end temperature 95 °C. The data were analysed using the software supplied with the instrument (PR.ThermControl v2.0.4, NanoTemper Technologies GmbH) and the Tm (for the ratio 350 nm/330 nm).

### 4.4. Apo-Enzyme Crystallisation and Structure Solution

Crystals of apo-*Oh*Lys, the wild-type, not the kexB variant, were grown in 96-well MRC crystallisation Plates^TM^ (Molecular Dimensions Ltd.), set up by a Mosquito Nanodrop crystallisation robot (Molecular Dimensions Ltd.). A total of 150 nl of protein was mixed with 150 nl of mother liquor solution. Crystals grew in 0.1 M MIB system (malonic acid, imidazole, boric acid), with pH 4.0 and 25% Peg 3350, corresponding to conditions B1–B4 of the PACT premier^TM^ screen (Molecular Dimensions Ltd.). Crystals were cryoprotected in a mother liquor solution, incorporating 25% glycerol prior to flash freezing in liquid nitrogen. Diffraction data were collected at the European Synchrotron Radiation Facility beamline ID23-1, at 100 K to 1.5 Å resolution.

All computations were carried out using programs from the *CCP4* suite [29], unless otherwise stated. Data were processed with MOSFLM [30,31], and scaled and merged with AIMLESS. The apo structure was solved by molecular replacement, using PHASER [32], with the coordinates of a human mutant lysozyme (PDB code: 1gft) as a search model. The starting model was improved manually using COOT [33], alternating with cycles of REFMAC [34]. The structure was validated using MOLPROBITY [35] prior to deposition.

### 4.5. Ligand Complexes Crystallization and Structure Solution

Screening for ligand complexes was carried out with the supposedly inactive mutants, E35A and D51A, both of which contained the kexB dipeptide insertion. E35A itself crystallised readily in INDEX screen no. 7 (Hampton Research) in Falcon 24 well plates, producing large, well-diffracted crystals, while it proved impossible to obtain crystals of the D51A mutant. Co-crystallisations of E35A were set up with chitobiose, chitotriose, chitotetraose, chitopentaose and chitohexaose, and were successful with chitobiose, chitotetraose, and chitohexaose. Crystals were harvested and frozen, and data were collected at the Diamond Light Source beam line IO4 for all three co-crystals. They were all isomorphous with those of the apo-enzyme, which was therefore used as the starting model. There was electron density in the active site of all three structures, but the density did not span the active site, and was restricted to the −1, −2 and −3 subsites, for a maximum of three monosaccharides. As the three structures were so similar, only that resulting from the chitohexaose sample was fully refined. It is assumed that the “inactive” E35A mutant had retained a low level of activity, sufficient to hydrolyse the longer oligosaccharides during complex formation and/or crystal growth. 

For both the apo enzyme and the ligand complex, data collection and refinement statistics are given in Table 1. Structural figures were drawn with CCP4mg [36].

### 4.6. Patents

The patent application “Lysozymes” (WO 2012/035103) [26] was based on the protein engineering work described in this paper.

## Figures and Tables

**Table 1:** Crystallographic statistics.

Data Collection ^(a)^		
Data set	Apo-Native	Mutant Ligand complex
Beamline	I02	I04
Wavelength (Å)	0.9795	0.9795
PDB code	6T5S	6T6C
Space group	P2_1_2_1_2_1_	P2_1_2_1_2_1_
Cell parameters (Å)	a = 36.97 b = 54.90 c = 65.32	a = 37.05 b = 55.01 c = 65.83
Resolution range (Å)	42.02-1.5 (1.53–1.50)	42.21-1.25 (1.27–1.25)
Number of reflections	109904	304278
Unique reflections	21950	38043
Monomers in asymmetric unit	1	1
Completeness (%)	99.8 (97.1)	99.9 (99.1)
<I/σ(I)>	12.3 (1.9)	13.4 (2.8)
CC_1/2_^(b)^	0.998 (0.646)	0.998 (0.535)
Multiplicity	5.0 (3.2)	8.0 (4.5)
R_meas_^(c)^	0.082 (0.637)	0.111 (1.013)
**Refinement statistics**		
Fraction of free reflections	0.050	0.050
Final R_cryst_	0.116	0.108
Final R_free_	0.166	0.134
R.m.s. deviations from ideal geometry (target values are given in parentheses)		
Bond distances (Å)	0.015(0.013)	0.018(0.013)
Bond angles (°)	1.74 (1.64)	2.19 (1.71)
Average main chain B values (Å^2^)	12.5	9.1
Average side chain B values (Å^2^)	16.1	13.3
Average B values for Ligand (Å^2^)	-	25.5
Molprobity score	1.85	1.94
Ramachandran favoured (%) ^(d)^	95.16	96.03
Ramachandran outliers (%) ^(d)^	0.0	0.0
Clash score	6.34	7.77

^(a)^ values in parentheses correspond to the highest resolution shell. ^(b)^ CC_1/2_ values for Imean are calculated by splitting the data randomly in half. ^(c)^ R_meas_ is defined as Σ √(N/N-1)|I - <I>|/Σ I, where I is the intensity of the reflection. ^(d)^ Ramachandran plot analysis was carried out by MOLPROBITY [21].

**Table 2:** The structures of GH22 lysozymes in the PDB and their similarity to *Oh*Lys.

Species	Common Name	PDB	Resn. Å	RMS Å	No. Cα
*Oncorhynchus mykiss*	Rainbow trout	1lmq	1.6	0.78	125
*Bos taurus*	Cow	2z2f	1.5	0.86	125
*Homo sapiens*	Human	133l	1.77	0.87	125
*Pelodiscus sinensis*	Chinese soft-shelled turtle	2gv0	1.9	0.92	125
*Tachyglossus aculeatus*	Australian echidna: Spiny anteater	1jug	1.9	0.93	124
*Phasianus colchicus*	Ring necked pheasant	1ghl	2.1	0.94	125
*Coturnix japonica*	Japanese quail	2ihl	1.4	0.95	124
*Mus musculus*	Mouse	4yf2	2.1	0.97	123
*Canis lupus familiaris*	Dog	1qqy	1.85	1.02	122
*Gallus gallus*	Chicken	3lzt	0.94	1.05	125
*Anas platyrhynchos*	Duck/Mallard	5v8g	1.2	0.98	125
*Colinus virginianus*	Virginia quail: Northern bobwhite	1dkj	2	1.09	125
*Equus caballus*	Horse	2eql	2.5	1.14	123
*Bombyx mori*	Domestic silk worm	1GD6	2.5	1.17	112
*Meleagris gallopavo*	Turkey	135l	1.3	1.18	125
*Musca domestica*	House Fly Lys1	2fbd	1.9	1.19	108
*Antheraea mylitta*	Silkworm	1IIZ	2.4	1.27	112
*Numida meleagris*	Helmeted guidea fowl	1hhl	1.9	1.31	125
*Musca domestica*	House Fly Lys2	3cb7	1.9	1.4	111
*Meretrix lusoria*	Bivalve	3ab6	1.78	2.33	80
*Tapes japonica*	Bivalve	2dqa	1.6	2.36	81

**Table 3:** The number of charged residues and pI for four representative lysozymes. Histidines have been excluded from the positive set.

	*Oh*Lys	Bovine	House Fly	HEWL
No. Residues	126	129	122	129
Negative Charge: Asp and Glu	20	15	9	9
Positive Charge: Arg and Lys	13	14	10	17
Ratio (negative/positive)	1.5	≈1	≈1	0.5
Total Charged	33	29	19	26
Theoretical pI	5.1	6.5	7.7	9.3

**Table 4:** Thermostability, as examined by nanoDSF.

	T_m_ (°C) at pH 4.7	T_m_ (°C) at pH 8.0
*Oh*Lys-KexB wt	65.2	55.4
R50T	59.8	53.3
K60R Y61W	64.8	54.2
S78P	66.9	57.4
D90A	69.0	64.0
I97V	62.7	54.3
Y108V	69.7	63.8
K124R	67.4	54.1

## Supplementary Materials

Supplementary Materials can be found at https://www.mdpi.com/1422-0067/20/22/5531/s1.

## Abbreviations

CAZy	Carbohydrate-Active enZYmes Database
GH	glycoside hydrolase
HEWL	HEWL: hen egg white lysozyme
NAG	*N*-acetylglucosamine
NAM	*N*-acetylmuramic acid
*Oh*Lys	*Opisthocomus hoazin* lysozyme
