# Supplementary material for: The C-Type Lysozyme from the upper Gastrointestinal Tract of Opisthocomus hoatzin, the Stinkbird
Source: Int J Mol Sci. 2019 Nov 6;20(22):5531. doi: 10.3390/ijms20225531 (PMC6887759; doi:10.3390/ijms20225531)
Supplement: Supplementary file 1 [file ijms-20-05531-s001.pdf]

International Journal of
Molecular Sciences

MDPI

Article

The C-Type Lysozyme from the upper Gastrointestinal Tract of *Opisthocomus hoatzin*, the Stinkbird

Supplementary Material

Supplementary Table S1. Sequence identifiers for the avian GH22 proteins in Figure 5. The common names are coloured according to the subfamilies described in Figure 5.

Common name (as shown in Figure 5)	Source organism scientific name (as shown in database)	Database reference	Cluster size (> 1)
Adelie penguin	Pygoscelis adeliae	SWISSPROT_A0A093NTL5	2
American crow	Corvus brachyrhynchos	SWISSPROT_A0A091FMC9	
Band-tailed pigeon	Patagioenas fasciata monilis	SWISSPROT_A0A1V4KD54	
Bare-faced curassow	Crax fasciolata	SWISSPROT_Q7LZQ3	
Barn owl	Tyto alba	SWISSPROT_A0A093FKQ0	
Blue-fronted parrot	Amazona aestiva	SWISSPROT_A0A0Q3WUF9	
Chicken	Gallus gallus	PDB_4MWN_A	63
Chinese bamboo partridge	Bambusicola thoracicus	SWISSPROT_B8YK69	
Collared flycatcher	Ficedula albicollis	SWISSPROT_U3JJC5	
Copper pheasant	Syrmaticus soemmerringii	SWISSPROT_P81711	
Crimson horned pheasant	Tragopan satyra	SWISSPROT_Q7LZI3	
Dalmatian pelican	Pelecanus crispus	SWISSPROT_A0A091SL88	
Duck (1)	Mus musculus	PDB_5VJQ_I	3
Duck (2)	Anas platyrhynchos	SWISSPROT_P00705	
Duck (3)	Anas platyrhynchos	SWISSPROT_P00706	
Egyptian goose	Alopochen aegyptiaca	SWISSPROT_P84496	
Emu	Dromaius novaehollandiae	SWISSPROT_G3XDT7	
Flamingo	Phoenicopterus ruber ruber	SWISSPROT_A0A091UEV3	
Gould's finch	Erythrura gouldiae	TREMBL_A0A3L8S3P1	
Great crested grebe	Podiceps cristatus	SWISSPROT_A0A094LH48	
Green junglefowl	Gallus varius	SWISSPROT_B8YK73	
Grey crowned crane	Balearica regulorum gibbericeps	SWISSPROT_A0A087VFU1	
Grey francolin	Francolinus pondicerianus interpositus	SWISSPROT_B8YK71	
Helmeted guineafowl	Numida meleagris	PDB_1HHL_A	2
Hoatzin	Opisthocomus hoazin	SWISSPROT_Q91159	
Japanese quail	Coturnix japonica	PDB_2IHL_A	2

Kalij pheasant	Lophura leucomelanos	SWISSPROT_P24364	
Lady Amherst's pheasant	Chrysolophus amherstiae	SWISSPROT_P22910	
MacQueen's bustard	Chlamydotis macqueenii	SWISSPROT_A0A091KHW5	
Northern fulmar	Fulmarus glacialis	SWISSPROT_A0A093JHL2	
Ostrich	Struthio camelus australis	SWISSPROT_A0A093HKR1	
Pekin duck	Anas platyrhynchos	PDB_5V92_A	
Plain chachalaca	Ortalis vetula	SWISSPROT_P00707	
Reeves's pheasant	Syrmaticus reevesii	SWISSPROT_P24533	
Ring-necked pheasant	Phasianus colchicus	PDB_1GHL_A	4
Rock dove	Columba livia	TREMBL_A0A2I0LIU5	2
Turkey	Meleagris gallopavo	PDB_2LZ2_A	6
Turkey vulture	Cathartes aura	SWISSPROT_A0A091LB50	
White-tailed eagle	Haliaeetus albicilla	SWISSPROT_A0A091PE21	
White-throated tinamou	Tinamus guttatus	SWISSPROT_A0A099Z0V9	
Wood duck	Aix sponsa	SWISSPROT_Q7LZQ2	
Zebra finch	Taeniopygia guttata	SWISSPROT_B5KFT9	

Table S2. Variants produced (all were active in the OD drop assay) The Hoatzin to HEWL mutations are shown in red, with 3 key changes in bold.

Mutations	Aim
kexB WT (*1aK+*1bR)	Potential cleavage site.
**R50T**	**Broader pH activity profile**
**Y61W**	**Substrate specificity**
V32A	Improved stability
D37N	Alkaline stability
K60R	Glycation susceptibility
K60R+Y61W	K60R: glycation susceptibility; Y61W: specificity.
K67R	Glycation susceptibility
S78P	Improved stability
E88T	Alkaline stability
**Y108V**	**Substrate specificity**
K124R	Glycation susceptibility
G19N+E21T	Addition of N-glycosylation
H34N	Addition of N-glycosylation
H34W	Improved stability
E41N	Alkaline stability
E41R	Alkaline stability
G47P	Improved stability
G74A	Improved stability
D89Q	Alkaline stability
D90A	Broader pH activity profile
K92A	Glycation susceptibility
K96R	Glycation susceptibility
I97V	Substrate specificity
A101P	Improved stability
K124S	Reduced glycation, improved stability
K124T	Glycation susceptibility, improved stability
